# Dielectrophoresis Prototypic Polystyrene Particle Synchronization toward Alive Keratinocyte Cells for Rapid Chronic Wound Healing

**DOI:** 10.3390/s21093007

**Published:** 2021-04-25

**Authors:** Revathy Deivasigamani, Nur Nasyifa Mohd Maidin, M. F. Mohd Razip Wee, Mohd Ambri Mohamed, Muhamad Ramdzan Buyong

**Affiliations:** Institute of Microengineering and Nanoelectronics, Universiti Kebangsaan Malaysia, Bangi 43600, Selangor, Malaysia; p101094@siswa.ukm.edu.my (R.D.); p103661@siswa.ukm.edu.my (N.N.M.M.); m.farhanulhakim@ukm.edu.my (M.F.M.R.W.); ambri@ukm.edu.my (M.A.M.)

**Keywords:** dielectrophoresis, fibroblast, keratinocyte, Clausius–Mossotti factor, electrical stimulation technique

## Abstract

Diabetes patients are at risk of having chronic wounds, which would take months to years to resolve naturally. Chronic wounds can be countered using the electrical stimulation technique (EST) by dielectrophoresis (DEP), which is label-free, highly sensitive, and selective for particle trajectory. In this study, we focus on the validation of polystyrene particles of 3.2 and 4.8 μm to predict the behavior of keratinocytes to estimate their crossover frequency (*f_XO_*) using the DEP force (*F_DEP_*) for particle manipulation. MyDEP is a piece of java-based stand-alone software used to consider the dielectric particle response to AC electric fields and analyzes the electrical properties of biological cells. The prototypic 3.2 and 4.8 μm polystyrene particles have *f_XO_* values from MyDEP of 425.02 and 275.37 kHz, respectively. Fibroblast cells were also subjected to numerical analysis because the interaction of keratinocytes and fibroblast cells is essential for wound healing. Consequently, the predicted *f_XO_* from the MyDEP plot for keratinocyte and fibroblast cells are 510.53 and 28.10 MHz, respectively. The finite element method (FEM) is utilized to compute the electric field intensity and particle trajectory based on DEP and drag forces. Moreover, the particle trajectories are quantified in a high and low conductive medium. To justify the simulation, further DEP experiments are carried out by applying a non-uniform electric field to a mixture of different sizes of polystyrene particles and keratinocyte cells, and these results are well agreed. The alive keratinocyte cells exhibit *N_DEP_* force in a highly conductive medium from 100 kHz to 25 MHz. 2D/3D motion analysis software (DIPP-MotionV) can also perform image analysis of keratinocyte cells and evaluate the average speed, acceleration, and trajectory position. The resultant *N_DEP_* force can align the keratinocyte cells in the wound site upon suitable applied frequency. Thus, MyDEP estimates the Clausius–Mossotti factors (CMF), FEM computes the cell trajectory, and the experimental results of prototypic polystyrene particles are well correlated and provide an optimistic response towards keratinocyte cells for rapid wound healing applications.

## 1. Introduction

Chronic wounds, which are injuries that have not matured through the normal cure phase, are visible for much longer than a month. Patients with diabetes are at risk of experiencing chronic wounds. An enormous majority of people with chronic open wounds usually have other substantial health issues. Around 400 million individuals globally live with diabetes [1]. In developed countries, the lifetime risk of diabetes is 45–64 years old. In 2030, developing countries are predicted to have 82 million more people above 65 years old with diabetes compared with 48 million people in developed countries [1]. The availability and quality of wound care are the major problems faced by patients with chronic wounds to recover faster. The absence of availability of advanced wound care results in amputations and a lack of work performance [2]. Generally, the human skin consists of successive layers. The epidermis, which is the epidermal layer keratinocyte membrane and the outermost layer of the skin, serves as a body shield to the outside world and acts as the first line of defense [3]. The keratinocyte is the building block of the skin epidermis and plays a crucial role in the proliferative wound healing process. The inner layer is the dermis, which consists of fibroblasts that are responsible for the synthesis of extracellular matrix (ECM) which supports wound migration [4]. The electrical stimulation technique (EST) addresses wound critical factors for example, reducing bacterial infection, improving capillary density and local perfusion, boosting wound oxygenation, promoting granulation and fibroblast development, and accelerating wound healing [5].

The alternating current in the EST techniques, with its benefits and drawbacks, is given in Table 1 [6,7,8]. EST therapy requires electrical transmission through wound tissues, which is usually done through electrodes. EST is tested specifically for diabetic foot wounds and pressure, venous, and vascular ulcers and shows promising effects on the rate of wound healing and recovery. Thus, the dielectrophoresis (DEP) method is more suitable for wound care, which falls under an alternating current by using the theory of the electrical kinetics model, is used [9]. The DEP technique is best suited for particle and cell manipulation using *F_DEP_*, which utilizes dielectric polarization. DEP is reliable and has good sensitivity and selectivity [10,11,12]. In 1951, Pohl first coined the word “Dielectrophoresis”, which is the phenomenon in which the alternating current of a nonuniform electric field is applied. The force on one charge will differ from the force on the other charge, resulting in a net force on the particle. The force is known as the *F_DEP_* [13]. DEP can be used for the manipulation, transport, separation, and sorting of various types of particles [14]. DEP is widely applied in the fields of medical diagnosis, drug discovery, cell therapy, and particle filtration [15]. The polystyrene particles have stable physicochemical properties and are used in the biochemical field because their size distribution is analogous to cells, including bacteria (2–4 µm) [16], red blood cells (7–8 µm) [17], liver cells (20–30 µm), and several cancer cells (10–30 µm) [18]. Moreover, polystyrene particles can provide potential factors for the manipulation of biomedical particles [15,19]. The keratinocyte cells are isolated from the human epidermis and have an average diameter of 7.96 μm approximately. However, the dermal layer of fibroblast cells is around 49 μm in size. The size of polystyrene particles is (2.5:10.2) µm decreased compared to standard keratinocyte and fibroblast cells, respectively, for effective particle manipulation and visualization. The polystyrene particle is subjected to EST to manipulate and synchronize the dispersed particle to the desired location.

The poor chronic wound heal scenario motivates us to incorporate a solution of DEP in diabetes rapid wounds healing. The DEP technique is used to manipulate and synchronize cells on human skin from the scattered position to the target location for the rapid healing of wounds. The keratinocyte cells play a crucial role in tissue repair through the EST. The prolonged application of EST can cause discomfort and even skin burns [20]. The aim of this study is therefore to investigate the short-term DEP effect of EST on wound healing.

Jonathan Cottet et al., in 2018, invented the new computational tool, MyDEP, which is a piece of stand-alone software (https://mydepsoftware.github.io, accessed on 11 January 2019) specially designed to study the dielectric particles and cell’s response to the AC electric field [21]. To investigate the electric field distributions and particle trajectory within the microchannel, a numerical model was solved using finite element analysis in COMSOL Multiphysics 5.5. (COMSOL Inc., Burlington, MA, USA). It is used to predict the DEP force (*F_DEP_*) exerted on the particles and cells [22]. Simulation results show how the cell trajectories change with the increasing frequency range. Microelectrodes with two-dimensional geometry were created for particle tracking. This study involved multiphysics such as AC/DC, fluid flow, and particle tracking for fluid flow. It allowed for the comparison of experimental and simulation results. As a result, the device’s performance should be predicted by these simulations [23,24]. The 2D/3D motion analysis software (DIPP-MotionV) software examines image tracking and analyses data from experimental DEP recorded video. As a result, the average speed, acceleration, and trajectory position of the cells are calculated.

In this present work, we have conducted the manipulation of 3.2 and 4.8 µm polystyrene particles by using DEP for the effective manipulation of alive keratinocyte cells. The results obtained from a polystyrene particle of 3.2 and 4.8 μm serve as a cross-reference to predict the behavior of keratinocytes to evaluate their crossover frequency (*f_XO_*) using the *F_DEP_* for cell manipulation. This is the significant reason for choosing the polystyrene particle. The prototypic polystyrene particles are widely used in the performance and quantitative evaluation of DEP systems to demonstrate the feasibility of the proposed highly sensitive biological cells. Furthermore, a numerical model involving MyDEP is performed, and its Clausius–Mossotti factors (CMF) and relevant particle orientation at various frequencies are determined. Additionally, the finite element method (FEM) is performed to track the particle mobility via applied input frequencies obtained from MyDEP. The numerically analyzed prototypic polystyrene particles of 3.2 and 4.8 μm have been practically tested. The particle trajectories are determined by applying an electric field to the tapered electrode based on its specific frequencies. In addition to target keratinocyte cells, the fibroblast cells are numerically investigated due to their different biophysical properties. In the case of keratinocyte and fibroblast cells, its corresponding frequencies were found from MyDEP and FEM. Thus, the evaluated frequencies from the MyDEP plot are well agreed with FEM computations for polystyrene particles, keratinocyte, and fibroblast cells. The prototypic polystyrene particles and alive keratinocyte cells are practically examined. The keratinocyte cells are analyzed in detail with DIPP-MotionV. Hence, this present study was attempted to manipulate alive keratinocyte cells for the development of quick wound healers based on the DEP technique.

## 2. Materials and Methods

The materials used during the experiment include fluorescent polymer microspheres, such as polystyrene beads of various sizes. The beads are purchased from Thermo Fisher Scientific (Fremont, CA, USA) and used in the preparation of all aqueous solutions. A mixture of 3.2 (20 µL) and 4.8 (20 µL) µm polystyrene particles in deionized (DI) water (60 µL) was prepared at a 1:1:3 ratio. Due to the low conductive suspension medium, it is directly applicable to DEP manipulation. Human Epidermal Keratinocytes (HEK) cells are cultured in a humidified incubator at 5% CO_2_ and 37 °C in Epilife suspension medium, which was purchased from (CTERM, HUKM, WP Kuala Lumpur, Malaysia).

This study focuses on the development of a rapid healer for chronic wounds. We have established the numerical simulation of polystyrene particles, keratinocyte, and fibroblast cells are analyzed using the MyDEP plot. The FEM analysis of polystyrene particles, keratinocyte, and fibroblast cells is executed in two different studies. The FEM findings include the electric field distribution, and the particle trajectories at different frequencies are analyzed. Furthermore, the 3.2 and 4.8 µm polystyrene particles and keratinocyte cells are manipulated by DEP using the fabricated tapered electrode. The keratinocyte cells are interpreted with DIPP-MotionV. Thus, the experimental results almost match the numerical data.

### 2.1. F_DEP_ Theory

The *F*_DEP_, acting on a dielectric particle suspended in a medium in an electrical field, can be approximated as Equation (1) [25]

(1) $$F_{DEP}=2\pi r_{ext}^{3}\varepsilon_{0}\varepsilon_{m}\;Re\;\left[f_{CM}\right]\nabla E_{rms}^{2}$$

where *ε_m_* is the medium relative permittivity surrounding the sphere, $r_{ext}$ represents the particle radius of polystyrene particles, keratinocytes, and fibroblast cell, *E_rms_* is the root-mean-square of the applied electric field, and *f_CM_* is the CMF of the cells describing their polarization with respect to the surrounding medium. In this case, the selective manipulation and the separation of particles with equivalent sizes depend on the real part of the CMF. *F_DEP_* is equivalent to the real part of the CMF, which is the in-phase attribute of the electrical polarization induced by the particle and associated with the spatial nonuniformity including its electric field [26].

(2) $$f_{CM}=\frac{\varepsilon_{p}^{*}-\varepsilon_{m}^{*}}{\varepsilon_{p}^{*}+2\varepsilon_{m}^{*}\;}$$

where $\varepsilon_{p}^{*}$ and $\varepsilon_{m}^{*}$ represent the complex permittivity of the particle and the medium, respectively, Equation (2) indicates that CMF depends on the electrical properties of the particle and the medium [27,28]. The CMF is determined based on the intrinsic dielectric properties of particles, which result in the *f_XO_* for each particle. The biological cell’s dielectric properties of capacitance and resistance reflect the plasma membrane composition, structure, cytoplasm, and nucleus of the cell. Each particle has different conductivity values. Thus, the polystyrene particles have sizes of 3.2 and 4.8 µm, which are calculated using the formula:

(3) $$\sigma_{p}=\sigma_{bulk}+\frac{2K_{s}}{r_{ext}}$$

where *σ_p_* indicates the particle’s electrical conductivity, σ*_bulk_* denotes the bulk conductivity and *K_s_* is the surface conductivity (i.e., polystyrene particles *σ_bulk_* = 1 × 10^−16^ S/m, *K_s_* = 2 × 10^−9^ S/m) [29]. Equation (3) indicates that the conductivity of particles is controlled by the surface conductivity and inversely proportional to the particle size. As such, the conductivity of the bulk becomes smaller than the conductivity of the surface and is ignored. The electrical conductivity values obtained for 3.2 and 4.8 µm polystyrene particles are 2.5 × 10^−3^ and 1.6 × 10^−3^ S/m, respectively.

The *F_DEP_* in Equation (1) is an easy and effective means of controlling particles. When the particle becomes more polarizable than the medium, the particle is believed to be attracted to the field as a positive DEP (*P_DEP_*). By contrast, whenever the particle becomes less polarizable than the medium that allows the particle to repel from high-field regions, this is known as negative DEP (*N_DEP_*). Theoretically, the *F_DEP_* direction should alternates at the crossover frequency (*f_XO_)* when the induced particle transforms from *P_DEP_* to *N_DEP_* or vice versa. When the net charge is set to *P_DEP_ = N_DEP_* = 0. As the particles are in a static position, the particle (*F_DEP_* = 0).

### 2.2. MyDEP for Numerical Simulations

MyDEP is a piece of computational software, a stand-alone application written in Java (Version v1.0.1). Zenodo (http://doi.org/10.5281/zenodo.2537957 (accessed on 11 January 2019)) evaluates the CMF of particles and cells. It is useful for predicting particle and cell responses to an electric field. The main criteria for estimating the cell mobility response require the detailed electrical and geometric parameters of the particles as an input specification [21]. This approach is used to analyze the CMF of the real and the imaginary phase of both particles and the suspension medium. The basic terminology to simulate the CMF plot requires the three modes of parameters from the literature, such as the (a) dielectric properties of the conductive medium, (b) input frequency range, and the (c) model of the cells or particle geometry and its dielectric properties. The characteristics of the used cells are listed in Table 2 [30,31,32]. The subsequent step is to establish the input frequency from 1000 Hz to 1 GHz.

Cell modelling is an essential step in MyDEP, in which polystyrene particles are a homogenous mixture, and the biological cells, such as keratinocyte and fibroblast cells, are classified as the single shell. The single-shell model consists of a cytoplasm covered by a cell membrane. The geometry of the particle or cells, such as shape and size, are considered. The polystyrene particle, also known as polymer microspheres, has a spherical shape. The dielectric properties of the particle include (a) electrical conductivity and (b) relative permittivity. These properties are used from the past study to simulate the following plot. The CMF to determine the orientation of the *F_DEP_* is calculated from Equation (1) using the MyDEP plot.

**Figure 1:**
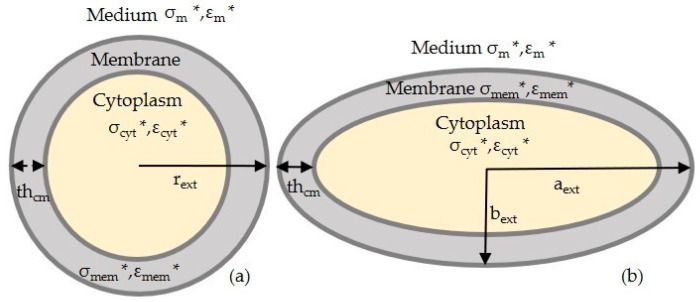
Geometry of the single-shell model: (**a**) keratinocyte cell and (**b**) fibroblast cell. An asterisk (*) indicating complex permittivity.

In general, permittivity is different for various biological tissues, providing unique dielectric signatures. The dielectric properties of keratinocyte cells are studied using dielectric spectroscopy [30]. Additionally, the same is numerically evaluated using the finite element method. Whereas the fibroblast cells (HL-60 cells) [33] are used as a reference, their dielectric parameters are determined from electrorotation measurements. The membrane and cytoplasm layer conductivity and permittivity are shown in Table 6. Relative to polystyrene particles of a single homogeneous model, the biological cells usually show more complex DEP behavior due to the multi-layered membrane and intracellular structures. In the present study, biological cells such as the keratinocyte and fibroblast cells are approximated as a single-shell model, and it is presented in Figure 1. The keratinocyte, fibroblast cells, and polystyrene particles with their respective Re [*f_CM_*] are evaluated. Those formulas are provided in the Supplementary Materials.

### 2.3. FEM Simulation

This numerical model was performed by a commercial FEM package (COMSOL Multiphysics 5.5, www.comsol.com, accessed on 14 November 2019). The establishment of particle manipulation with a tapered DEP microelectrode uses two key steps.

Study 1, the electrical current and the laminar flow interface are used. Electric current defines the electrical potential and it is conducted in a frequency domain (AC). The laminar flow is interlinked to the stationary study.
Study 2 involves the particle tracing for fluid flow interface. This interface relies on the results from Study 1. The coupling was achieved by interlinking the frequency domain solution of Study 1 to solve the particle trajectory with *F_DEP_*. The coupled equation system of Study 2 was solved using the acquired Study 1 solution; therefore, Study 1 and 2 are interlinked. A time-dependent study was used to support this analysis.

**Figure 2:**
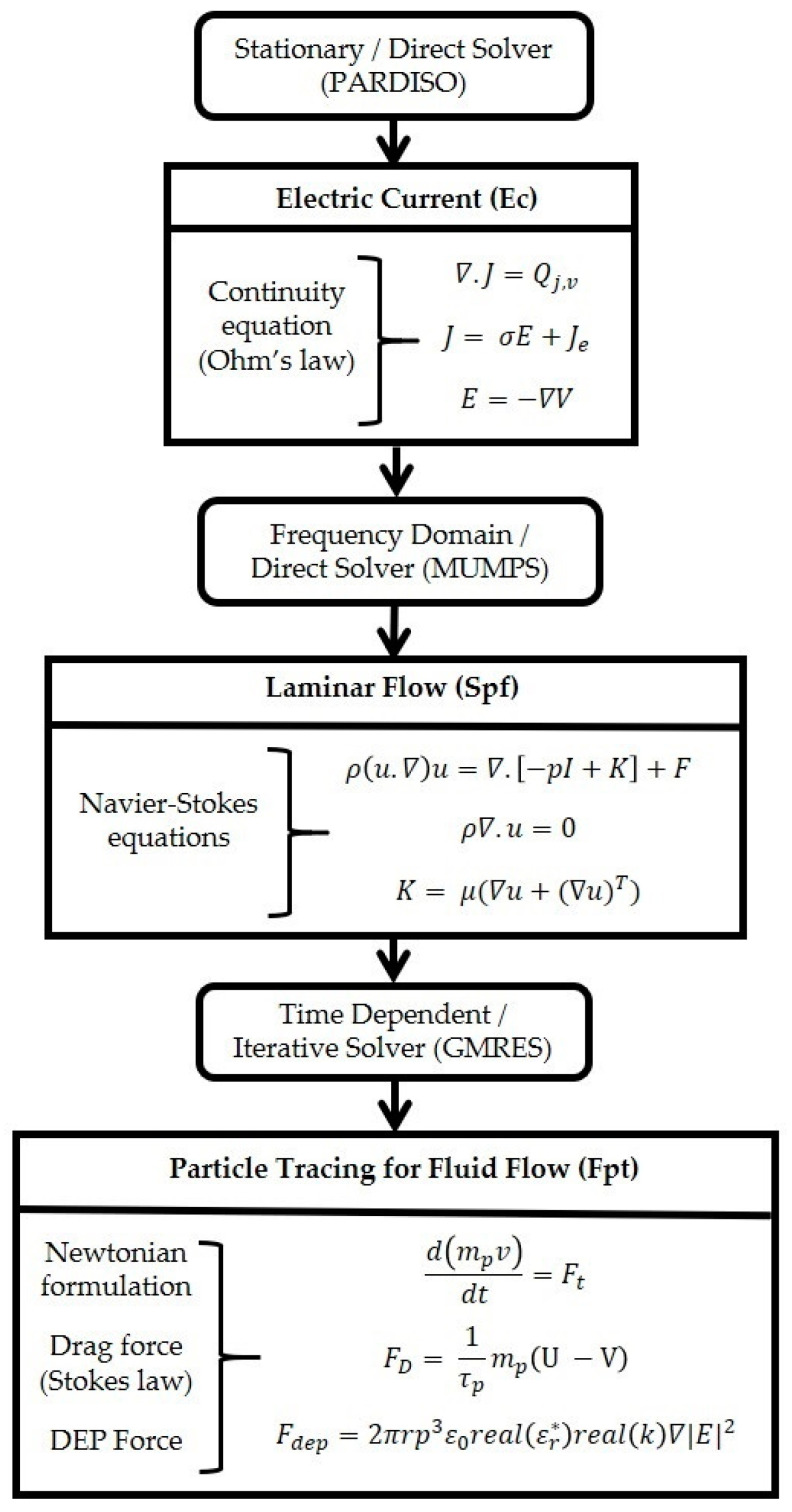
Flowchart with governing equations and solver for each interface.

The tapered electrode geometry is created in a two-dimensional model. We defined the material for the tapered electrode as aluminum, and for the fluidic channel we used low conductive DI water, while for the high conductive medium we used Dulbecco’s modified eagle medium/nutrient mixture F-12 (DMEM/F-12), and Dulbecco’s modified eagle medium (DMEM). The three kinds of the interface were chosen for particle trajectories in Figure 2 with its respective solver, and the governing equation is given. Firstly, the electric current interface solves a current conservation equation based on Ohm’s law. The continuity equation in Ec must be considered when handling stationary electrical currents in conductive media. The current conservation node adds the continuity equation for the electrical potential and provides an interface to define the electric conductivity (σ) as well as the relative permittivity (ε) for the displacement current and utilizes the equation in Ec. The initial values node acts as an initial condition for the electric potential (initial values = 0 V). In the electric insulation node, all boundaries are selected except electric potential edges and utilized boundary condition n.J=0, where no electric current flows into the boundary. In the electric potential node, set the input voltage V_0_ to +10 V and −10 V using boundary condition V = V_0_. The value of the potential is defined at the edges of an electrode to solve electric potential.

Secondly, the laminar flow interface is used to calculate the pressure fields for the flow of a single-phase fluid. The Navier–Stokes equations are for the conservation of momentum and the continuity equation for mass conservation in Figure 2, Spf being solved by the laminar flow interface. The fluid properties node adds the momentum and continuity equations and defines the density and viscosity of the fluid according to the conductive medium. The initial values for the velocity field u and the pressure p are specified zero. The pressure point constraint may be used to specify the pressure level. The relative pressure value is determined by specifying the boundary condition for pressure P = P_0_, where (P = 0 Pa), P_0_ represents the absolute pressure. The pressure for fluid flow is 0 Pa, considered to be negligible. The wall node contains a set of boundary conditions, U = 0, that describe fluid-flow conditions in the stationary study. By default, the no-slip wall condition is used.

Finally, the particle tracing for fluid flow interface is used to compute the motion of particles in a background fluid. Particle motion is driven by drag and *F_DEP_*, its wall condition stated as Freeze in V = V_c_. Thus, after contact with the wall, the particle positions no longer change. Besides, specified particle properties such as charge number and particle velocity based on the Newtonian formulation in Fpt are given in Figure 2 [34]. The particles released from the grid node are used for locating the particle position in the tapered electrode and its initial coordinates for two types of particles/cells are in Table 3.

During the experiment, the mixture of a particle is used in the region of interest (ROI). However, for a clear vision of FEM analysis, we have considered the 3.2 µm polystyrene particle or keratinocyte in the left and the 4.8 µm polystyrene particle or fibroblast in the right of the tapered electrode using boundary condition Q = Q_0_ and V = V_0_. The polystyrene particles, keratinocyte, and fibroblast cells are released from the grid node in the ROI. Furthermore, drag force has been calculated in Fpt using the Stokes law, which specifies the velocity field and material properties for particle motion in a laminar flow. The *F_DEP_* equation expressed in Fpt Figure 2 and specified the particle and fluid properties. The *F_DEP_* node is used to exert a force on the particles. The force is specified via an electric field. The influence of the *F_DEP_* on the particles depends on the difference in permittivity between the particles and the fluid. When ε_p_ > ε_m_, the particles are attracted to regions where the absolute electric field is strong. When the ε_p_ < ε_m_, the particles are attracted to regions where the absolute electric field is weak.

Particle velocity is obtained from Drags law given in Fpt, shown in Figure 2. The drag law used and the rarefaction effects applied to the drag force determine the particle velocity response time, where τ_p_ is the particle velocity response time. The forces acting on each particle are verified from the external fields using a pre-solved electric field in the angular frequency method each time the solver takes a step. Later, chosen particles to affect the list, are used to apply the force to particle position. Additionally, piecewise polynomial recovery is used for specific particles. Added shell subnodes, due to a single shell, are used on specific particles. Added shell subnodes are due to a single-shell model of biological cells. In the case of polystyrene particles, no shell subnodes are added, and the particle is treated as a homogeneous sphere for computing *F_DEP_*.

**Figure 3:**
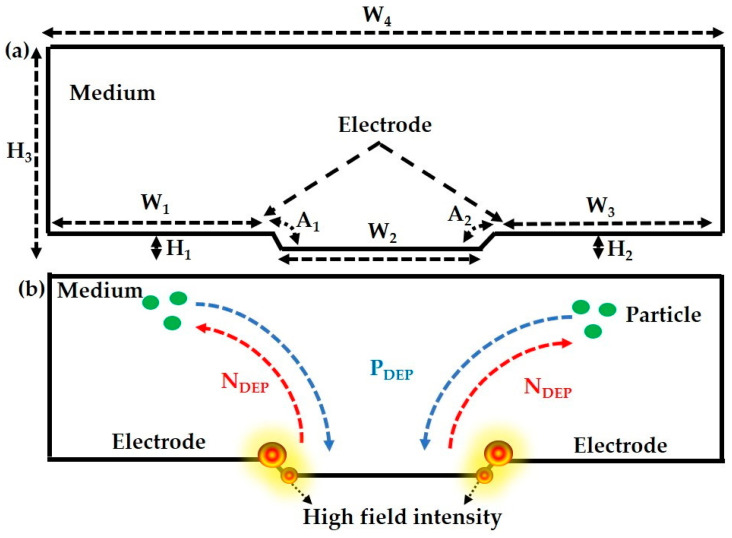
Tapered microelectrode geometry with specifications: (**a**) 2D geometry of a tapered microelectrode device; (**b**) illustration of a particle trajectory sequence in a tapered microelectrode.

The two-dimensional geometry of the modelling microelectrode device is presented in Figure 3a. Furthermore, the microelectrode device consists of an ROI that contains a structure of tapered electrodes of alternating polarity that regulates the particle trajectories. The fluid medium is considered an ROI, where the material is added as a droplet in the practical study. While in the DEP experiment, the particles or cells are dispersed to the top of the microelectrode surface using a droplet technique, the volume of the sample distribution is assumed to be similar to the FEM analysis. A non-uniform electric field is applied to the tapered electrode, resulting in higher field intensity on the top and bottom edges of the microelectrodes are presented in Figure 3b. In the case of *P_DEP_*, the input electrical field is applied, which in turn generates a lateral force in the microelectrode and causes the particles or cells to be drawn to high field intensity. While in *N_DEP_* the input field generates a lateral force, in the electrode this leads to the particles or cells in that region traveling up to the top of the electrode. The electrode dimensions, such as the width (W), height (H), an inclination of 75°, and the angles A_2_ that indicate the inclination of 15° in a rectangle, make the electrode tapered. The ROI has a total width (W_4_) of 280 µm and height (H_3_) of 80 µm and all the dimensions are presented in Table 4. The meshing element size is normal and the user-controlled sequence type is used throughout the simulation.

### 2.4. Fabrication of a Microelectrode

The tapered aluminum microelectrode arrays (TAMA) are fabricated using the CMOS processing approach and implemented on a Si substrate. The initial step requires a plasma-enhanced chemical vapor deposition (PECVD) method for the deposition of 1.15 µm SiO_2_ as an insulator layer upon the uppermost part of the Si substrate, followed by the deposition of a thin titanium/titanium nitride (Ti/TiN) adhesion layer with a thickness of 60/30 nm. Furthermore, the following step involves Ti/TiN deposition of a 4.0 m thick aluminum/silicon/copper layer (Al/Si/Cu-98 wt.%/1 wt.%/1 wt.%) using physical properties. To transfer the square array design to the Al/Si/Cu layer, UV curing for photoresist hardness is used. Finally, Al/Si/Cu is etched using an inductively coupled plasma (ICP) etcher for metal etching via an advanced plasma-resistant stripe. An additional resist tapered profile formation step is implemented before the final Al/Si/Cu etch step. The new combination of resistant profile process and etching technology provides the desired tapered angle of profile for microelectrodes. Resist plasma etching through reactive ion etching (RIE) and metal etching by ICP methods can be used together to develop a desired tapered microelectrode profile [35,36,37].

### 2.5. DEP Experimental Setup

**Figure 4:**
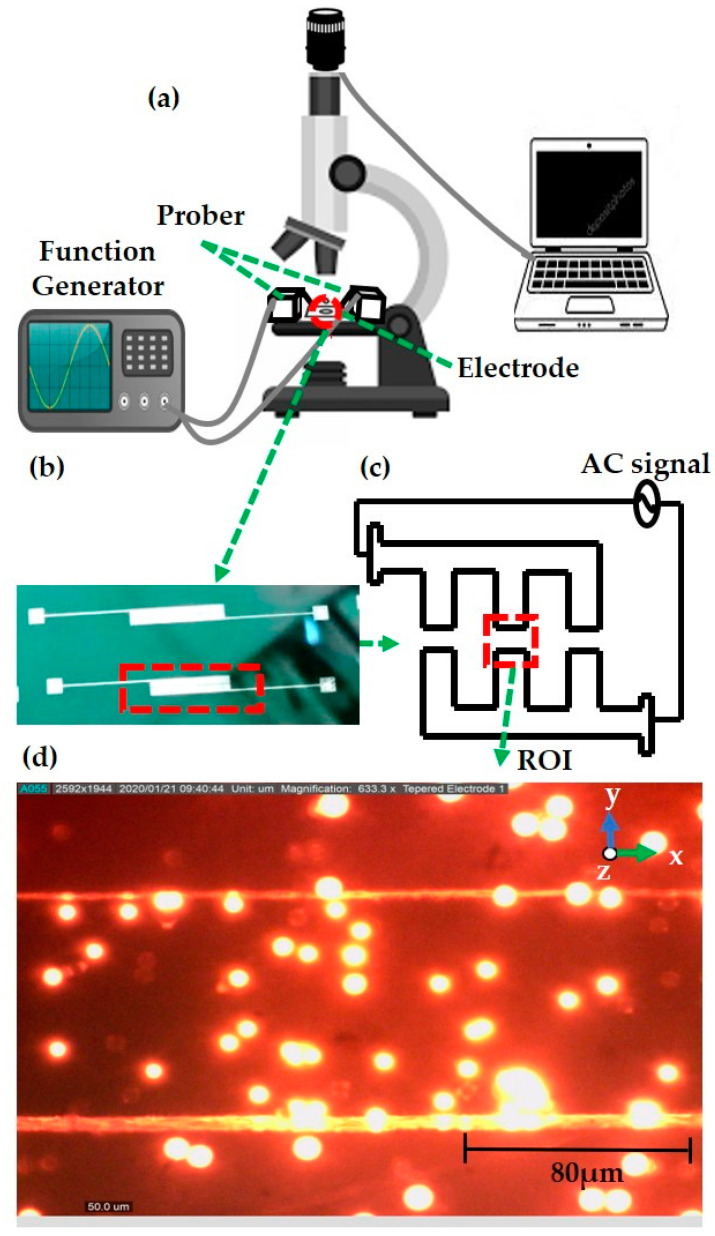
Overview of the dielectrophoresis (DEP) framework: (**a**) pictorial view of the experimental setup; (**b**) fabricated tapered electrode; (**c**) top view of the tapered microelectrode; (**d**) region of interest (ROI)—particle in a tapered electrode.

The pictorial perspective of the experimental design is illustrated in Figure 4a. The experimental manipulation process of polystyrene particles is carried out using a trinocular microscope (MOTIC-BA400) installed with a conventional microscope. An additional eyepiece microscope camera (Dino-Eye, AM7025X) is fixed to the eyepiece microscope for video recording. The Dino-Capture 2.0 software supports the image processing investigation to predict the particle DEP effect. The camera recorder is connected to the system to monitor and record the movement of particles, while the manipulation of keratinocyte cells is performed using a fluorescence microscope (OLYMPUS-BX53M). Visual monitoring of the keratinocyte cell DEP effect is supported by the cellSens standard software. The input sinusoidal electrical signals from a function generator (Teledyne LeCroy-WaveStation, 2022; 20 V peak to peak, 15 MHz) are directly connected to the prober, and its positive and negative terminals are linked to supply a voltage of different frequencies to the microelectrode.

A droplet of the prepared mixture of polystyrene particles or cultured keratinocyte cells (0.2–0.5 µL) is dispersed on the top of the tapered microelectrode surface later covered with a glass slip to visualize the *F_DEP_* behavior. Proper filter selection is the key to use successful fluorescence microscopy. Barrier filters are filters designed to suppress or block (absorb) the wavelengths of excitation and allow only selected wavelengths of emission to pass to the eye or other detector. In the polystyrene particles experiment, the green excitation filter is used, which covers an excitation wavelength range between 510 and 560 nm, while the keratinocyte cells are visualized in bright field mode.

The different magnification ranges are focused. Microelectrode identification is performed by initially viewing at 4×, 10×, and 40× for the visualization of enlarged particles. However, the electrode length is calibrated to 80 µm. In the case of polystyrene particles, a total of 22 tests are performed using the same input voltage of 10 voltage peak to peak (V_PP_) with the applied frequency in the range of 100–500 kHz, and 20 kHz increment steps are applied to the microelectrode for up to 30 s for every test run. In the keratinocyte cell experiment, 15 tests were conducted with 10 V_PP_ applied voltage at a frequency of 100–900 kHz, 1–25 MHz, 100 kHz, and 5 MHz, respectively, with progressive changes applied to the tapered electrode at the 30s per test. The illustrative view of the fabricated tapered electrode is indicated in Figure 4b. The overall top view of the TAMA layout is represented in Figure 4c and the region of interest (ROI, Figure 4d). The *F_DEP_* behavior is experimentally measured, and the lateral attraction or repulsion is observed at the ROI. The space gap on each side is 80 μm, and microelectrodes with a square array display 1100 × 1100 µm.

### 2.6. DIPP-MotionV Motion Analysis

2D/3D motion analysis software (DIPP-MotionV) investigates the image tracking and data analysis of keratinocyte cells. As an outcome, this analysis generates the average speed, acceleration, and trajectory position of the cells to be interpreted based on the experimental keratinocyte cell measurement movie. The motion analysis has been done using the desired recorded video from the experiment, and the step-by-step procedure for motion analysis is elaborated in the Supplementary Materials.

## 3. Results

### 3.1. Numerical Analysis by MyDEP

Simulation is widely used to define an optimized design to improve particle or cell capture. Thus, MyDEP is used to estimate the CMF and *f_XO_* of polystyrene particles of various sizes, keratinocytes, and fibroblast cells and in their conductive medium. Particles experience a *P_DEP_* or *N_DEP_* effect based on the polarity of the real part Re [fCM]*,* which varies from +1 to −1/2 and −3/4 for the imaginary part Im [fCM] [13]. Notably, the CMF is plotted only for the real part because it is the only contribution to DEP (the imaginary part relates to electrorotation, another AC electrokinetic effect). Table 5 includes the dielectric properties of low and high conductive media, such as DI water, DMEM/F-12, and DMEM. The effective dielectric values and sizes of 3.2 and 4.8 μm polystyrene particles, keratinocytes, and fibroblast skin cells are shown in Table 6.

#### 3.1.1. Modelling of Polystyrene Particles

**Figure 5:**
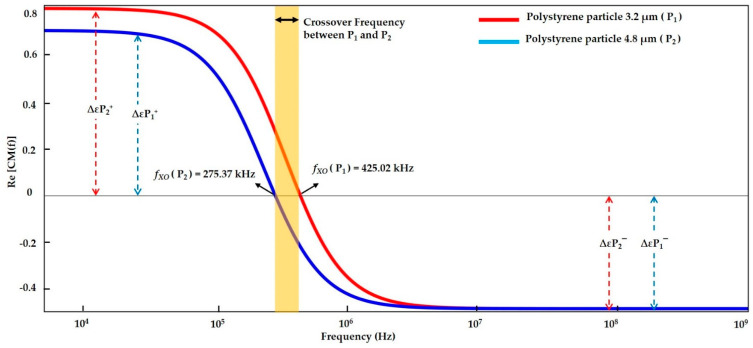
Clausius–Mossotti factors (CMF) of polystyrene particles with sizes of 3.2 and 4.8 µm.

Polystyrene particles are usually homogeneous in nature and spherical in shape. The polystyrene particles work well in DI water, a low conductive medium. MyDEP software is used to accurately model the resulting graph by incorporating Table 6 dielectric values. At zero baselines, the curves transition from *P_DEP_* to *N_DEP_* is recognized as *f_XO_*. The CMF curve of the polystyrene particles is shown in Figure 5. It shows that the above zero baselines correspond to a *P_DEP_* region, which is the movement of particles attracted to a high field intensity.

The particles are attracted to the electrode, whereas below zero baselines are considered to be an *N_DEP_* region, which means that the particle is repelled from a high field intensity, and the majority of the particles accumulate in the low-field intensity region. The total crossover range reflects that the mobility of particles within this range is static and lies dynamically between two *f_XO_*. The equilibrium state is reflected by *P_DEP_* = *N_DEP_* = 0 net charge. 

The polarization factors of the 3.2 and 4.8 μm polystyrene particles are observed to have *f_XO_* values of 425.02 and 275.37 kHz, respectively. The simulation findings show that the smallest size of polystyrene 3.2 μm particle tends to have a high *f_XO_* of 425.02 kHz. By contrast, the 4.8 μm polystyrene particle has a large particle size with a low *f_XO_* of 275.37 kHz.

#### 3.1.2. Modelling of Keratinocyte and Fibroblast Cells in a Low Conductive Medium

The basic internal characteristics for the epidermal layer keratinocytes and the dermal layer fibroblast cells for the MyDEP simulation to examine the CMF and the *f_XO_* of biological skin cells are summarized in Table 6. The manipulation of the cells is accomplished through the influence of dielectric properties and it can distinguish various cell types.

**Figure 6:**
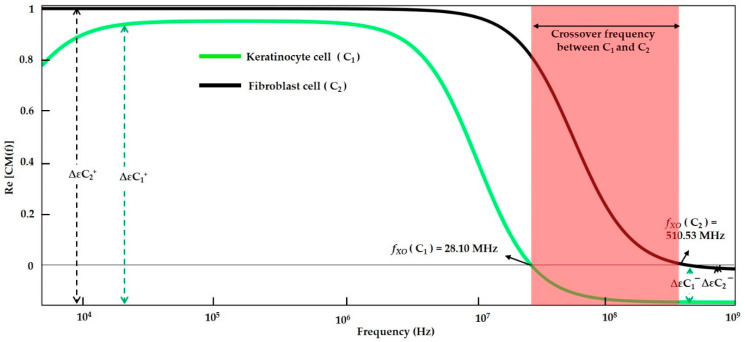
CMF result for epidermal layer keratinocyte and dermal layer fibroblast cells in low conductive medium.

The keratinocyte and fibroblast cell’s behavior to the frequency in a low conductive medium are presented in Figure 6. The *f_XO_* is indicated for the defined medium, and the transition from the *P_DEP_* to the *N_DEP_* regime is 28.10 and 510.53 MHz, respectively. The two cells can be manipulated between these frequencies based on their electrical properties. Thus, MyDEP analysis estimates that the small-sized epidermal keratinocyte cell has *f_XO_* at 510.53 MHz, and the fibroblast cell of the largest size tends to have *f_XO_* at 28.10 kHz. Each particle has a distinct atomic structure, creating different polarization factors for each particle. Through this design, the orientation of the *F_DEP_* is determined.

#### 3.1.3. Modelling of Keratinocyte and Fibroblast Cells in a High Conductive Medium

**Figure 7:**
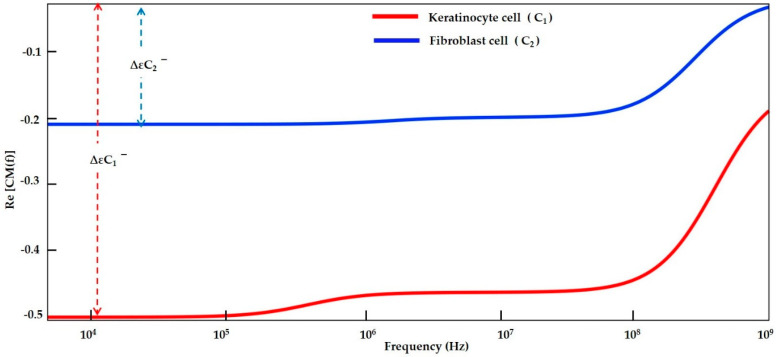
CMF plot for keratinocyte and fibroblast cells in dulbecco’s modified eagle medium/nutrient mixture F-12 (DMEM/F-12) and dulbecco’s modified eagle medium (DMEM) in high conductive medium.

The CMF curve of the keratinocyte and fibroblast cells in the high conductive medium of DMEM/F-12 and DMEM, respectively, are shown in Figure 7. It could theoretically and experimentally be proven in the literature that cells exhibit exclusively *N_DEP_* if suspended in highly conductive media. Therefore, the particles/cells can be repelled from surfaces by appropriate arrangements of electrodes that can easily be manipulated in free solution [42]. From the plot at all frequency ranges, the keratinocyte and the fibroblast cells experience *N_DEP_*. The cell is repelled from the high field intensity compared to the fibroblast cell, the keratinocyte cell experiences strong *N_DEP_* due to its high electrical conductivity value and the same is demonstrated in Section 3.2.4.

#### 3.1.4. Numerical Result Evaluation of Polystyrene Particles and Biological Skin Cells

**Figure 8:**
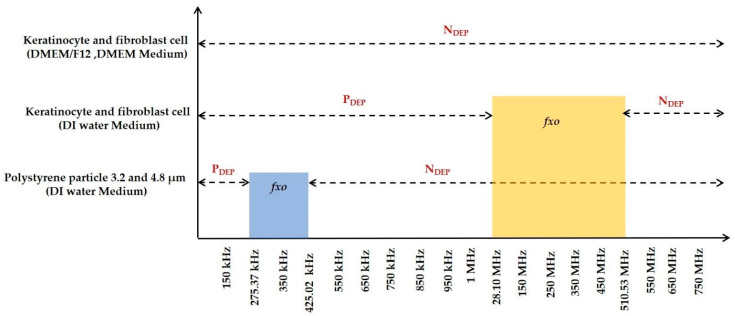
Performance analysis from the MyDEP simulation of polystyrene particles, epidermal layer keratinocyte, and dermal layer fibroblast cells.

The data from the MyDEP plot in Figure 5, Figure 6 and Figure 7 is analyzed and reported in Figure 8. The polystyrene particles with sizes of 3.2 and 4.8 µm in the low conductive medium have a *P_DEP_* in the range of 0 to 275.37 kHz, where the particles are assumed to be attracted to a high field intensity. By contrast, the particles greater than 425.02 kHz are *N_DEP_*, which causes the movement of particles repelled from high field intensity, whereas the polystyrene particle 4.8 μm is *f_XO_* in the range of 275.37 kHz and the polystyrene particle 3.2 μm is *f_XO_* at 425.02 kHz. Thus, the particles experience an equilibrium state and are almost static. The MyDEP plot is calculated based on its dielectric properties to estimate their particle mobility and orientation of the *F_DEP_*. Relative to the polystyrene particle, the keratinocyte and fibroblast cells in the low conductive medium have a *P_DEP_* response from 10 kHz to 28.10 MHz. *N_DEP_* is predicted to be above 510.53 MHz, and an *f_XO_* response is in a wide range of 28.10 MHz and 510.53 MHz for the keratinocyte and fibroblast cells, respectively. Similarly, for the keratinocyte and fibroblast cells in high conductive medium, DMEM/F-12 and DMEM are observed. At an entire frequency range from 10 kHz to 750 MHz, the keratinocyte and fibroblast cells exhibit *N_DEP_*. Based on the interpretation of MyDEP data, the predicted frequency is proven in the Section 3.2 FEM analysis. Similarly, the experiment of the keratinocyte cell in high conductivity medium and the obtained DEP behavior is demonstrated in Section 3.3.2.

### 3.2. FEM Analysis

The steady-state or stationary study and frequency domain (AC) are solved to evaluate the distribution of electric potential in the channel. A time-dependent study is done using a pre-solved solution from the frequency domain in an electric field. The particle trajectories of 3.2 µm polystyrene particle or keratinocyte and 4.8 µm polystyrene particle or fibroblast cells under the influence of DEP and drag forces are used to estimate particle tracing. The main idea of this simulation is based on these three different effects: When the applied frequency is fixed below the *f_XO_*, the particle or cells are displaced toward the region of maximum field intensity, which is referred to as *P_DEP_*. When the applied frequency is higher than the *f_XO_* magnitude, the particles are forced to move towards the regions of minimum field intensity, which is referred to as *N_DEP_*. At the *f_XO_*, the particles are in an equilibrium state.

#### 3.2.1. Electric Current

**Figure 9:**
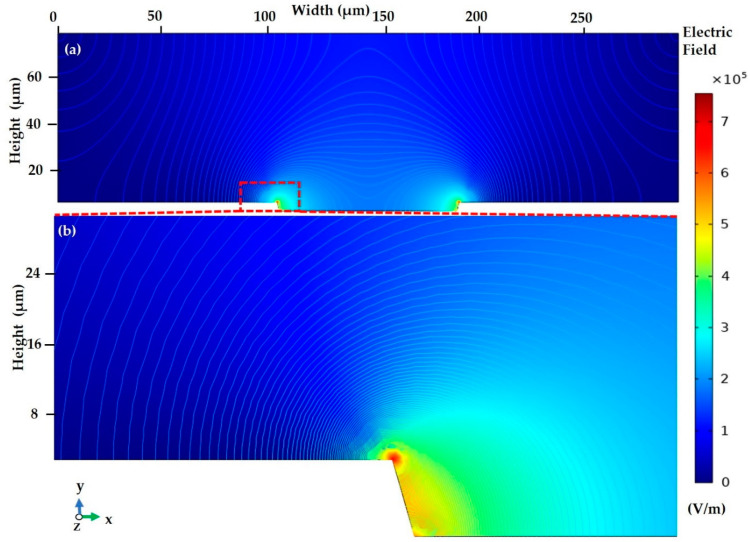
At stationary study: (**a**) spectral change in electrical field in the tapered microelectrode with contour plot; (**b**) the enlarged view of the high field intensity spot at the edges of the microelectrode.

Particle trajectory is controlled by the applied electric potential of 10 V to +10 V in the tapered electrode. Study 1 is used for the stationary and frequency domain, which solves the pressure, and AC electric potential. The geospatial electric potential of the microelectrode is indicated in Figure 9. In the initial step of the simulation, we have applied a non-uniform electric field to the electrode. The *F_DEP_* is acquired on the positive electrodes (+10 V) and the negative electrodes (−10 V) with the contour of an electric field in Figure 9a. The particle tracking is established on the respective frequency varies based on the stationary study of the tapered electrode edges with a high field intensity spot. This phenomenon leads to particle attraction or repulsion from the electrodes [43]. The tapered electrode edge with two high field intensity spots is illustrated in Figure 9b with its enlarged view.

#### 3.2.2. FEM of Polystyrene Particles

The FEM analysis for 3.2 and 4.8 µm polystyrene particles is implemented using a similar methodology as keratinocyte and fibroblast cells. Furthermore, the exploration of the polystyrene particles using FEM is elaborated as follows. The particle tracking for fluid flow (fpt) is computed, which generates three different cases of particle/cell analysis, and its relevant computation results are shown below.

**Figure 10:**
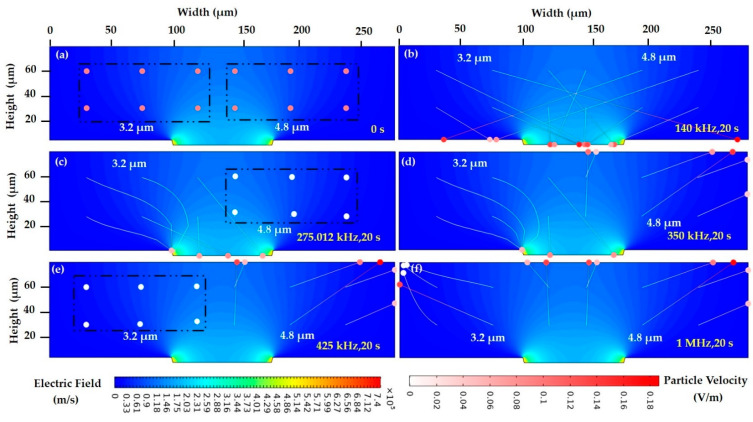
Particle trajectories at (**a**) the initial stage at 0 s with an initial particle velocity of 0 m/s, (**b**) 140 kHz, 20 s, 3.2 and 4.8 µm—*P_DEP_*, (**c**) 275.012 kHz, 20 s, 3.2 µm—*P_DEP_*, 4.8 µm—*f_XO_*, (**d**) 350 kHz, 20 s, 3.2 µm—*P_DEP_*, 4.8 µm—*N_DEP_*, (**e**) 425 kHz, 20 s, 3.2 µm—*f_XO_*, 4.8 µm—*N_DEP_*, and (**f**) 1 MHz, 20 s, 3.2 and 4.8 µm—*N_DEP_*. The color gradient of the trajectory line indicates the instantaneous particle velocity u ranging from 0 m/s (white) to 0.16 m/s (dark red). The contour and intensity of the electric field are indicated by the background blue color.

Case 1: The polystyrene particles of 3.2 and 4.8 µm experience *P_DEP_* at a low frequency and are attracted to a high field intensity spot, near the electrode edges. In Figure 10a, the particles at the initial step at 0 s in the microelectrode were spread uniformly in the ROI. Particle tracing with the applied frequency at 140 kHz is implemented, and the majority of cells move towards the high field and get attracted to the electrode in a lateral direction and experience *P_DEP_* with its instantaneous particle velocity, as is presented in Figure 10b. Thus, at 140 kHz, both the 3.2 and 4.8 µm polystyrene particles experience a significantly larger magnitude of *P_DEP_*, and their corresponding particle velocity is high, which reaches the designated tapered electrode within a short timescale. As a result of the strong *P_DEP_* response at a low frequency, the trajectory particle is very straight and outside the maximum intensity spot.

Case 2: The polystyrene particles acquire the equilibrium position in two separate *f_XO_*. However, in the intermediate frequency, the 4.8 µm polystyrene particles undergo *N_DEP_*, and the 3.2 µm polystyrene particles are attracted to high field intensity *P_DEP_*. At an applied frequency of 275.012 kHz, the 3.2 µm polystyrene particles get accumulated to high field intensity through lateral force, i.e., *P_DEP_*. The 4.8 µm particles are almost static with a velocity of 0 m/s, as is presented in Figure 10c. The *f_XO_* is achieved through *P_DEP_* = *N_DEP_* = 0 [44]. The intermediate frequency applied between the two *f_XO_* ranges at 350 kHz. Thus, the 3.2 μm polystyrene particles are attracted towards the high field intensity, *P_DEP_* and the 4.8 µm particles are repelled from the high field, and *N_DEP_* is presented in Figure 10d. Its particle’s orientation and migration are displayed in trajectory lines. The 3.2 µm polystyrene particle is very close to the crossover frequency range of 425 kHz, resulting in a slightly lower magnitude of *P_DEP_* response than at 140 kHz, which resembles the CMF plot in Figure 5. Hence, Figure 10c at 275.012 kHz and Figure 10d at 350 kHz, which depict *P_DEP_*, show curved trajectories near the maximum intensity spot. In Figure 10e, the 4.8 µm particles at 425 kHz are shifted to a low field spot; meanwhile, the 3.2 µm polystyrene particles are stagnant without large movement with a particle velocity of 0 m/s.

Case 3: Both cells are repelled from high field intensity, and the particle exhibits *N_DEP_*. At 1 MHz, both particles got repelled to high field intensity, i.e., *N_DEP_*. Thus, the 3.2 and 4.8 µm polystyrene particles reach the low field spot with a short-time interval due to their different particle properties. Thus, FEM simulation estimates that 4.8 µm particles are attracted more rapidly than 3.2 µm polystyrene particles. In Figure 10f, the particle that appears in the white color indicates 0 m/s velocity, and the red color indicates 0.16 m/s. The 4.8 µm polystyrene particles are attracted rapidly with their velocity of approximately 0.06 m/s compared to the 3.2 µm polystyrene particle (three particles in 0 m/s). 

#### 3.2.3. FEM of Biological Cells in a Low Conductive Medium 

The particle trajectory in the low conductive medium, for keratinocyte and fibroblast cells, experiences three different cases.

**Figure 11:**
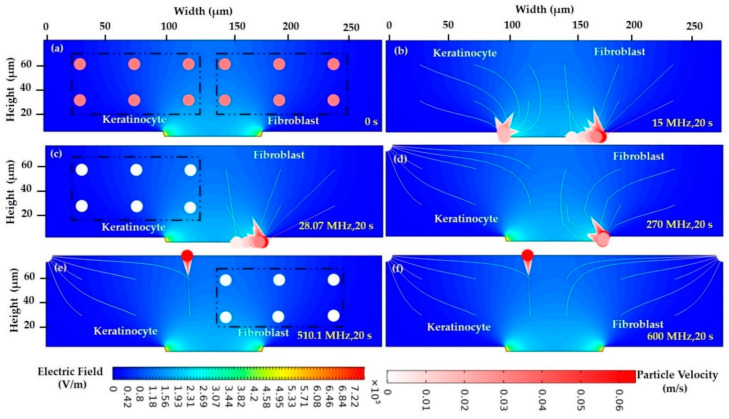
Particle trajectories in low conductive medium at (**a**) the initial stage at 0 with an initial particle velocity of 0 m/s, (**b**) 15 MHz, 20 s, keratinocyte and fibroblast—*P_DEP_*, (**c**) 28.07 MHz, 20 s, keratinocyte—*P_DEP_*, fibroblast—*f_XO_*, (**d**) 270 MHz, 20 s, fibroblast—*P_DEP_*, keratinocyte—*N_DEP_*, (**e**) 510.1 MHz, 20 s, fibroblast—*f_XO_*, keratinocyte—*N_DEP_*, and (**f**) 600 MHz, 20 s, keratinocyte and fibroblast—*N_DEP_*. The color gradient of the trajectory line indicates the instantaneous particle velocity u ranging from 0 m/s (white) to 0.6 m/s (dark red). The contour and intensity of the electric field are indicated by the background blue color.

Case 1: The keratinocyte and fibroblast cells experience *P_DEP_* and are attracted to high field intensity, near the tapered electrode. At 15 MHz, the cells were distributed evenly at 0 s in the ROI, as shown in Figure 11a. The majority of cells move towards a high field intensity and get attracted to the electrode in a lateral direction and experience *P_DEP_* at 20 s, as shown in Figure 11b. Keratinocytes cells have a greater force and are more deflected and their instantaneous particle velocity is presented with different color levels.

Case 2: The *f_XO_* for both cells is attained in a specific frequency and the cell has no response and is in a static position, while in the intermediate frequency, the keratinocyte cell undergoes *N_DEP_*, and the fibroblast cell moves towards the electrode, attracted to high field intensity, i.e., *P_DEP_*. At 28.07 MHz, the fibroblast cell is attracted to high field intensity through lateral force, i.e., *P_DEP_*, whereas the keratinocyte cell is stable and inactive with a particle velocity of 0 m/s, as presented in Figure 11c. In this condition, *P_DEP_* = *N_DEP_* = 0, the keratinocyte cell of 270 MHz (in-between range of 28.07 and 510.1 MHz). Thus, the fibroblast cells are moved towards the high field intensity, *P_DEP_*, and the keratinocyte cells are repelled from the high field, *N_DEP_*, as presented in Figure 11d. Its cell’s orientation and migration are displayed in trajectory lines. In Figure 11e, most of the keratinocyte cells are attracted to low field intensity, *N_DEP_*. Meanwhile, the fibroblast cells are static in an equilibrium position with a particle velocity of 0 m/s at 510.1 MHz.

Case 3: Both cells are repelled from high field intensity, and the cells encounter *N_DEP_*. At 600 MHz, the keratinocyte and fibroblast cells are shifted to the low field intensity with instantaneous particle velocity, as presented in Figure 11f. The keratinocyte and fibroblast cells are repelled to high field intensity experiences, *N_DEP_*.

#### 3.2.4. FEM of Biological Cells in a Highly Conductive Medium

The DEP technique is most suitable for low conductive samples. Biological cells such as keratinocyte and fibroblast cells are usually cultured in high conductive media (DMEM/F-12, DMEM). The difficult aspect is that living cells in a low conductive medium are very sensitive. However, the alive keratinocyte cells in a high conductive medium are experimentally manipulated and exhibit *N_DEP_*.

**Figure 12:**
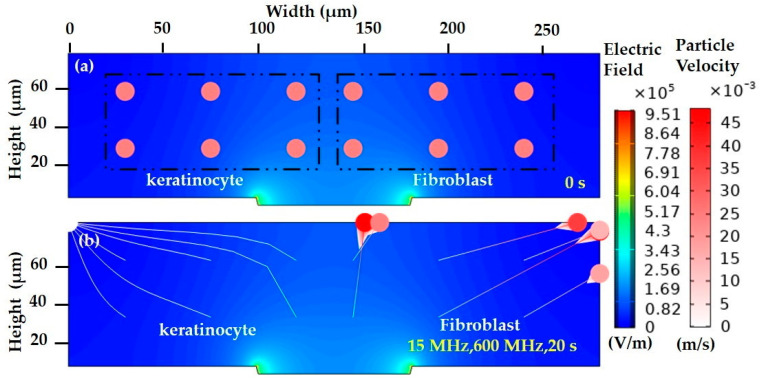
Particle trajectory in high conductive medium at (**a**) the initial stage at 0 with an initial particle velocity of 0 m/s and (**b**) 15 and 600 MHz, 20 s, keratinocyte and fibroblast—*N_DEP_*. The color gradient of the trajectory line indicates the instantaneous particle velocity u ranging from 0 m/s (white) to 46 m/s (dark red). The contour and intensity of the electric field are indicated by the background blue color.

Case 1: In a high conductive medium, the particle trajectory response is indicated in Figure 12. At the early stage of 15 and 600 MHz, in 0 s, the cells are placed equally in the ROI, as shown in Figure 12a. In the 20 s, the keratinocyte and fibroblast cells are migrated to the low field intensity, *N_DEP_*. The fibroblast cells, which are greater than the keratinocytes in size, have a greater force and are more deflected in a short time compared to the keratinocyte cell. The keratinocyte cell has an instantaneous particle velocity of 0 m/s, whereas the fibroblast has a velocity of 46 m/s, as presented in Figure 12b.

### 3.3. DEP Experiment

**Figure 13:**
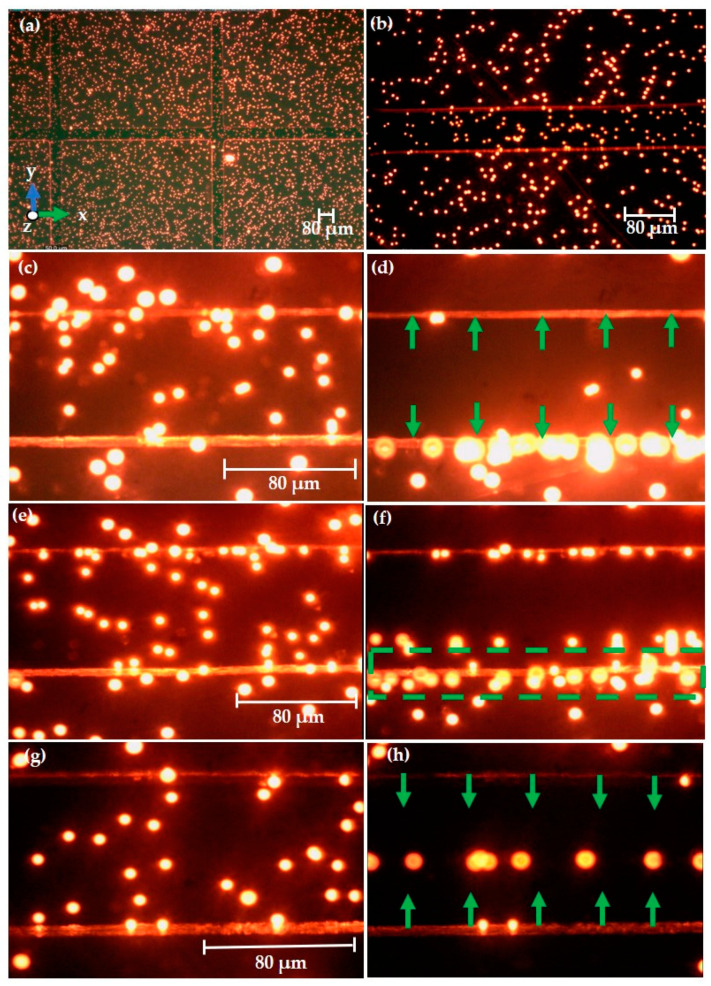
Fluorescence microscopy observations of 3.2 and 4.8 µm polystyrene particles in deionized water conductive medium for effective *F_DEP_*: (**a**) 4× and (**b**) 10× magnifications of microelectrode with polystyrene particles; (**c**) initial 0 V_PP_ at 200 kHz; (**d**) final 10 V_PP_–*P_DEP_* force at 200 kHz; (**e**) initial 0 V_PP_ at 350 kHz; (**f**) final 10 V_PP_–intermediate frequency at 350 kHz; (**g**) initial 0 V_PP_ at 460 kHz; (**h**) final 10 V_PP_–*N_DEP_* force at 460 kHz.

**Figure 14:**
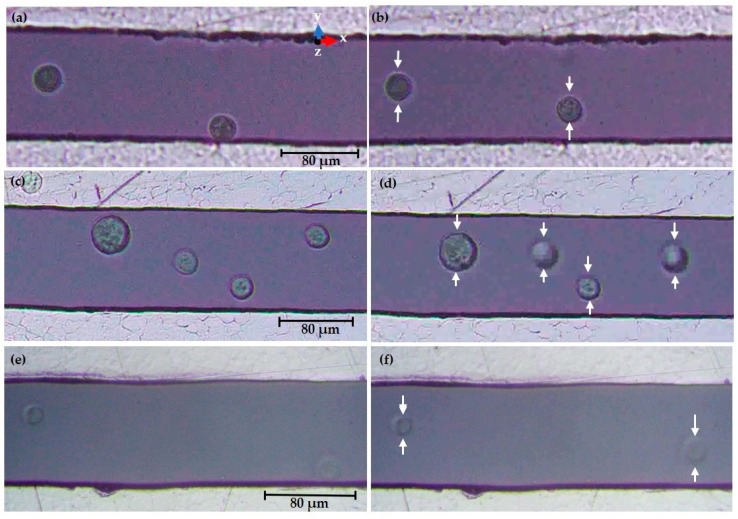
Fluorescence microscopy observations of keratinocyte cells in DMEM/F-12 conductive medium for effective *F_DEP_*: (**a**) initial 0 V_PP_ at 300 kHz; (**b**) final 10 V_PP_–*N_DEP_* at 300 kHz; (**c**) initial 0 V_PP_ at 800 kHz; (**d**) final 10 V_PP_–*N_DEP_* at 800 kHz; (**e**) initial 0 V_PP_ at 15 MHz; (**f**) final 10 V_PP_–*N_DEP_* force at 15 MHz.

The manipulation of DEP at two separate drive directions is illustrated in Figure 13. This experimental research is intended to estimate the *F_DEP_* at its respective frequency and constant applied voltage of varying sizes of polystyrene particles. *F_DEP_* is categorized as *P_DEP_* and *N_DEP_*. *P_DEP_* is the lateral attraction to the upper surface of the microelectrodes, and *N_DEP_* is the lateral repulsion between the two microelectrodes. This research consequently introduces perspective advancements for the lateral manipulation of 3.2 and 4.8 μm polystyrene particles by using tapered DEP microelectrodes for the accumulation of two various particles of different sizes. Besides, alive keratinocyte cells are also manipulated in their respective medium. This research is considered to be essential for the further development of a rapid wound healer and is shown in Figure 14.

#### 3.3.1. Experimental Observation of Polystyrene Particles

The magnified views of microelectrodes at 4× and 10× are presented in Figure 13a,b, respectively. For future measurement of the DEP effect, the 40× magnification is used for improved visualization of particles. The capturing action is performed as per the following time frame. Initially, almost all the microelectrode measuring areas are filled with suspended particles. The particle movement is tracked for 5 s after applying a desired electrical field and observing for 20 s. The applied field is turned off, and the particle mobility is eventually observed for 5 s. Figure 13c displays the voltage without being applied, i.e., 0 V_PP_ indicates that no electric field is applied. The particle is in a scattered state, and the alternating current of 10 V_PP_ is applied. The sudden particle drift is observed when the voltage is applied, and the variable frequency from 100 to 260 kHz is monitored. Particles attempt to attract to the high-field intensity electrode region, and most of the particles are not located in the middle of the low-field intensity region due to the more polarized particles than the conductive medium. The *F_DEP_* is dominant and experiences the *P_DEP_* effect. In Figure 13d, the resulting particle is observed to encounter a *P_DEP_* by tuning the voltage of 10 V_PP_ and the frequency of 200 kHz.

Figure 13e implies the particle without the application of an electrical field, i.e., at 0 V_PP_, the particle is distributed in the region of interest (ROI). After applying a voltage of 10 V_PP_, the frequency range from 260 to 420 kHz is tuned, and the magnitude of *P_DEP_* to *N_DEP_* is equal at this alternating current at *f_XO_*. the charges are in a balanced state, and the particles are static. In that case, no dominant *F_DEP_* is observed. Likewise, the equilibrium line location is determined by the respective values of the two counteracting *F_DEP_* values, which are regulated externally by the electrical signal applied to the metal electrode. The applied voltage of 10 V_PP_ and frequency set at 350 kHz reveal the particle in an intermediate state a 3.2 µm particle is *P_DEP_* and a 4.8 µm particle experiences *N_DEP_* in Figure 13f. In other words, the highlighted region suggests that, in this frequency range, the particle adheres to a two-force (*P_DEP_* and *N_DEP_*). Figure 13g depicts the particle in a dispersed state without an applied field (0 V_PP_). The applied field and frequency are 10 V_PP_ and 460 kHz, respectively.

The *F_DEP_* drives cells in liquid due to the interaction between a polarizable particle and a non-uniform electrical field. A close observation reveals that the polystyrene particles build up between the microelectrode. The phenomenon is known as *N_DEP_*. This result confirms that the *F_DEP_* is dominant. The applied voltage of 10 V_PP_ at a 460 kHz frequency indicates that the majority of particles are repelled from the electrode, and particles are aligned in the middle, as indicated in Figure 13h. Table 7 simplifies the data of the DEP experiment and the subsequent particle mobility depending on the input frequency. The 3.2 and 4.8 µm polystyrene particle manipulation is compatible with simulated data. Hence, good performance is obtained.

#### 3.3.2. Experimental Observation of Keratinocyte Cells

The droplet of 0.5 μL keratinocyte cells is added to the microelectrode at the early stage and focuses on the cells in the 20× magnification lens. The movement of the cell is tracked and observed in the time frame of 20 s after applying the desired electrical field. Figure 14a displays the alive keratinocyte cells at a random position even without voltage being applied, i.e., 0 V_PP_ at the 300 kHz frequency. When the alternating current of 10 V_PP_ at 300 kHz is applied, the keratinocyte cells are eventually shifted to the low field intensity spot at the middle of the electrode encounter *N_DEP_* and it is included in Figure 14b.

The keratinocyte cells are situated in the ROI without applying an electrical field, as represented in Figure 14c, i.e., at 0 V_PP_, 800 kHz. The frequency was tuned to 800 kHz and applied the input voltage of 10 V_PP_. The keratinocyte cells are repelled from the high field intensity and the cells accumulate in the middle and experience *N_DEP_*, as shown in Figure 14d. If the exposure to the electric field is too long, the cells move out of the ROI. 

Similarly, in Figure 14e, the cells are located in a scattered location at 0 V_PP_ and 15 MHz. The keratinocyte cell experiences the repulsion force and accumulates in the middle of the ROI after applying the voltage, as presented in Figure 14f. Throughout this experiment, in all of the above three cases, keratinocyte cells experience *N_DEP_* at 300 kHz, 800 kHz, and 15 MHz due to a high conductive medium. This result confirms that the *F_DEP_* is dominant and that the *F_DEP_* is dependent on the intrinsic dielectric properties and is consistent with the MyDEP simulated data. Due to the Joule heating effect, an improvement in the sinusoidal input signal is not desirable, however. This phenomenon affects the physical and biological properties of the test sample. The potential damage is due to the structure and molecular interactions of the cell membrane, affecting stress-related biological functions and activating membrane-bound proteins [30].

When a high voltage of 15–25 V_pp_ was applied, the production of such strong electric fields heats the surrounding medium due to the Joule heating, which was observed in the form of the electrothermal effect [45]. When an external electrical field was applied across an electrode, Joule heating creates temperature gradients near the electrode, which induces permittivity and conductivity gradients. A bulk electrical force has been generated and results in the interaction between the electric field and the gradient, which causes fluid motion. To dominant DEP force in a highly conductive medium, the DEP experiment on cell migration is carried out at 10 V_pp_ to prevent the electrothermal effect. Lower voltages were applied on samples with higher conductivities to avoid electrolysis and bubble formation. 

It is essential to consider when operating at highly conductive samples. Initially, the electrothermal force and fluid velocity are both strongly voltage dependent on the applied voltage. Furthermore, a high temperature could cause sample degradation and other undesirable electrothermal effects. When frequencies greater than 100 MHz were used for DEP manipulations, the cells experienced a more substantial diminishment due to the thermal effect. As the frequency rises, so does the dissipated energy, which is absorbed by the media and cells. Thermal stroke may have an impact on cell genetic expression [46].

### 3.4. DIPP-MotionV Motion Analysis for Keratinocyte Cells

As discussed above in Section 3.3.2, DIPP-MotionV motion analysis is used to analyze experimentally manipulated keratinocyte cells at three different frequencies. The purpose of this analysis is to determine the average cell velocity, acceleration, and position of the coordinate. 2D/3D motion analysis software (DIPP-MotionV) enables the target cells to be labelled. The initial and final positions of the cells, as well as the speed and acceleration of cell movement, will be interpreted. The coordinate position is useful for determining the movement of cells with a linear or non-linear velocity. The data are shown in Table 8.

At 300 kHz in Figure 14b, two cells are labelled as C_1_ and C_2_ and followed the tracking procedure as indicated in the Supplementary Materials. The interpreted data indicate that C_2_ moves quickly after the applied electrical field and accumulates at the center position of the ROI, while C_1_ does not have a large movement due to the cell already located near the low field region. The trajectory is based on XY coordinates. The cells have a linear velocity. For keratinocyte cells at 800 kHz in Figure 14d, four cells are marked as C_1_, C_2_, C_3_, C_4_ to be tracked. If an electrical field is applied, the cells near the electrode move to the middle of the ROI and exhibit *N_DEP_*. After long field exposure, the cells moved out of the ROI. The XY coordinates reveal that cells have a velocity in linear. In Figure 14f at 15 MHz, the cells are assigned as C_1_ and C_2_ and the movement from the initial to the final position is not exceeded by a large distance because the two cells are almost near the low field intensity area and experience the linear movement towards the electrode. 

## 4. Discussion

The results from FEM and the MyDEP are illustrated in Table 9. The comparison list from Table 7 shows that FEM frequency values are very close to the experimental and MyDEP data of 3.2 and 4.8 μm polystyrene particles. Even though a polystyrene particle is homogenous and exhibits a different DEP response than cells, the potential benefits of polystyrene particles are demonstrated as an experimental proof-of-concept for cell migration before the use of keratinocyte cells in this practical study [10]. Our aim is to validate the model with numerical analyses and ensure that the experimental setup is working as expected for cell synchronization. Experimental data for polystyrene particles are considerably remarkable frequency responses with simulation based on tabulated values. The results from MyDEP and FEM of 3.2 and 4.8 μm polystyrene particles are almost similar in *P_DEP_,* *f_XO_*, and the *N_DEP_* ranges. In high conductive medium, keratinocyte and fibroblast cells use DMEM/F-12 and DMEM, respectively. With the evaluated MyDEP plot at all possible frequencies, the cells experience only *N_DEP_* and the cells are attracted to the low field intensity, which is proven from both the estimated MyDEP and FEM and practically manipulated the alive keratinocyte cells. Additionally, from the literature, it is cross-verified and reported that for high conductive medium usually the *N_DEP_* is mostly possible [47]. Thus, for keratinocyte and fibroblast cells in both high and low conductive medium, numerical evaluated data are well matched with the FEM analytical values. Moreover, Table 10 reported the obtained theoretical results for keratinocyte and fibroblast cells with the available experimental data in the literature. In 2007, Broche et al. have investigated the early detection of oral squamous cell carcinoma (cancer detection). By applying a frequency signal between 5 and 10 kHz, DEP can be used to isolate cancer cells from more normal epithelial cells. Cancer cells are collected, since cells trapped by DEP are retained and it can also provide an initial determination of the presence of oral cancer cells. They investigated the transformed normal skin keratinocytes and oral keratinocytes from a tumor [48]. The novelty in this research is that living skin keratinocyte cells are manipulated using the DEP technique for wound healing application for diabetic sufferers by applying an electric field.

Increases in medium conductivity above a certain threshold may induce immunity in the DEP forces response, resulting in no negative or positive DEP detected between 100 Hz and 100 MHz. Thus, in the high conductive medium, the *P_DEP_* is not detected in this study [46]. It is crucial when working with living microorganisms, although using low-conductivity buffers frequently results in a high mortality rate of microorganisms due to osmotic stress [1]. Another method for trapping targets from a high conductivity medium is to use *N_DEP_*, which repels particles toward local electric field minima [42]. Although all cells experience *N_DEP_* in a high conductivity medium, we consider only a selected frequency to produce a stronger *N_DEP_* force, causing the cells to migrate faster for keratinocyte cell alignment in the wound site for rapid recovery [49].

This study attempted to use EST in DEP wound healing applications. Keratinocyte cells appear early in the skin and secrete both platelet-derived growth factor (PDGF) and transforming growth factor-beta (TGF-β), which stimulates dermal fibroblasts in scattered positions. In this stage, the DEP microelectrode is activated, and the keratinocyte cell can migrate to the low field region and align the keratinocytes in the wound site based on the applied frequency. Further, the cells are synchronized, allowing for wound contraction using DEP-assisted wound therapy. Hence, the *N_DEP_* force is used to manipulate each keratinocyte cell from a random location to the wound site, causing them to align at the target position and expedite wound closure. 

## 5. Future Perspective for Wound Healing Application

Diabetic chronic wounds are predicted to be a great issue in the future. Therefore, more research is needed to find an effective solution for rapid chronic wound diagnosis. In this work, the DEP technique is implemented to develop a rapid wound healer. In this research, the polystyrene particles are used to predict the migration behavior of keratinocyte cells. The low conductive medium is adequate for particle mobility for polystyrene particles. Although, it is crucial to use a highly conductive medium in the DEP experiment due to the effect of joule heating and electrolysis. In this study, keratinocyte cells are cultured in the highly conductive suspension medium and manipulated using the DEP technique. As a result, due to the presence of high salt concentration in the culture medium, most of the frequencies experience an *N_DEP_* effect. Subsequently, the culture medium would be replaced by a low conductivity DEP buffer medium, which will be less polarizable than the cells providing the necessary osmotic balance to the cells, which will be used as a DEP buffer medium to enhance *P_DEP_* strength [55]. A low conductivity DEP buffer is used to produce *P_DEP_* and trap cells.

The wound healing stages are further elaborated below to understand the mechanism. A wound in the skin transfers to a deeper layer. The initial step is the activation of the platelet and the formation of a blood clot. Epidermis layer cells such as neutrophils, macrophages and lymphocytes provide cell immunity and simulate the growth factors. The mechanism of keratinocyte and fibroblast cells produces angiogenesis and myofibroblast for proliferation and activation. Further keratinocytes and fibroblast cells are generated and dispersed at different locations during the proliferation process in the epidermal and dermal layers. The wound is rebuilt with new tissue made of collagen and ECM [56].

At this stage, the DEP microelectrode activates to speed up the alignment of keratinocytes and fibroblasts in their respective layers for faster wound recovery. Cell mobility and synchronization to epidermal and dermal layers are achieved via the implementation of DEP. This improves the wound contraction and reorientation of the keratinocyte cells to the target site for wound care. The alignment duration has been improved based on the DEP, and cell restorative tissue recovers rapidly without delay. The final product is then assumed to be a quick wound healer using an EST, mimics the natural current of injury, and accelerates the healing process.

## 6. Conclusions

The interactions of molecules either repulsive or attractive are primarily dependent on the applied frequency. In this research, the molecular interactions of both polystyrene particles of 3.2 and 4.8 µm and alive keratinocyte cells are explored. The computational modelling of prototypic polystyrene particles of 3.2 and 4.8 μm is used to predict the behavior of keratinocyte cells. The MyDEP technique is compatible with FEM analysis with its calculated crossover frequency. The numerical method estimates that 28.10 and 510.53 MHz are the *f_XO_* of the keratinocyte and fibroblast cells, respectively. The prototypic 3.2 and 4.8 μm polystyrene particles’ *f_XO_* range is 425.02 and 275.37 kHz, respectively. Besides, the FEM is used to evaluate the electric field intensity as well as the particle trajectory. The particle trajectory is carried out by controlling the input frequency, and its respective particle mobility was observed for polystyrene particles in the low conductive medium. The trajectories of keratinocyte and fibroblast cells have been estimated in high and low conductive media. Experimental testing with polystyrene particles of different sizes is in good agreement with the research based on simulation results. From this study, the keratinocyte cells are successfully manipulated in a highly conductive medium and the same validated with numerical model MyDEP and FEM and analyzed with DIPP MotionV for rapid wound healer application. 

## Figures and Tables

**Table 1:** Alternating current features in the electrical stimulation technique (EST).

Current Type	Kind of EST	Function of Current	Briefing of Techniques	Pros and Cons in Wound Healing
Alternating current (AC)	(i) Transcutaneous electrical nerve simulation (TENS) (ii) Dielectrophoresis (DEP) (Present work)	(i) Bidirectional distribution of charged particles and constant movement changes in the direction of the charge flow. (ii) Alternate polarity	(i) Electrode alternates the polarity continuously with each cycle. Thus, no charge production under the electrode is observed. (ii) In medical care, TENS therapy is used to minimize chronic and acute pain.	PROS: AC is more effective than DC in minimizing the wound volume or size. CONS: Low-frequency AC is not broadly used due to the shortage of polarity in the treatment of the wound healing process.

**Table 2:** Characteristics of keratinocyte and fibroblast cells.

Key Features	Keratinocyte Cell	Fibroblast Cell
Layer	Epidermal layer	Dermal layer
Primary task	Provide protective properties	Synthesizes extracellular matrix (ECM) and collagen
Structure	Auto-renewing stratified squamous epithelium	Large, smooth and elongated
Shape	Cuboid to spherical	Spindle like ellipsoid
Cell model	Two-dimensional	Three-dimensional
Nucleus	Nucleus-free	Flat and oval
Size	7.96 µm	49 µm

**Table 3:** Particle release from grid with its initial coordinates.

Particle Position	Particles/Cells	Initial Coordinates	
Grid-1 (Electrode left)	3.2 µm polystyrene particle/keratinocyte cell	q_x_,_0_ (30,45,130) µm	q_y_,_0_ (30,30,60) µm
Grid-2 (Electrode Right)	4.8 µm polystyrene particle/fibroblast cell	q_x_,_0_ (150,45,270) µm	q_y_,_0_ (30,30,60) µm

**Table 4:** Tapered microelectrode dimensions.

Symbol	Values (µm)
W_1_	100
W_2_	80
W_3_	100
W_4_	280
A_1_	75°
A_2_	15°
H_1_	4
H_2_	4
H_3_	80

**Table 5:** Dielectric properties of high and low conductive medium.

Conductive Medium	Electrical Conductivity (σ_p_) (S/m)	Relative Permittivity (ε_p_)	Reference
Deionized Water	2 × 10^−4^	78	[38]
Dulbecco’s Modified Eagle Medium/Nutrient Mixture F-12	2.3	80	[39]
Dulbecco’s Modified Eagle Medium	1.5	80	[40]

**Table 6:** Specifications of polystyrene particles with sizes of 3.2 and 4.8 µm, epidermal layer keratinocytes, and dermal layer fibroblast cells.

Particle/Cells	Diameter (µm)	Electrical Conductivity (σ_p_) (S/m)	Relative Permittivity (ε_p_)	Reference
3.2 µm Polystyrene particle	3.2	2.5 × 10^−3^	2.56	[38]
4.8 µm Polystyrene particle	4.8	1.6 × 10^−3^	2.56	[38]
Epidermis layer (keratinocyte)	5.97, 5.97, 11.95	Cytoplasm—0.12	50	[41]
		Cell membrane—1 × 10^−6^	9.04	
Dermis layer (fibroblast)	70, 70, 7	Cytoplasm—0.75	75	[40]
		Cell membrane—0.0047	53	

**Table 7:** Data interpretation from the experimental study and the resulting mobility of the polystyrene particles dependent on the applied input frequency.

Response	Input Frequency	Particle Mobility
*P_DEP_* (Lateral attraction)	*f* < 260 kHz	Attracted to the top surface of microelectrode—high field intensity
Intermediate frequency (lateral attraction and repulsion)	260 kHz *< f* < 420 kHz	Attraction to high intensity—3.2 µm Repulsion to low intensity—4.8 µm
*N_DEP_* (lateral repulsion)	*f* > 420 kHz	Repelled in between the microelectrode—low field intensity

**Table 8:** Average values of speed, acceleration and coordinate position of keratinocyte cell trajectory.

Frequency	Cells	Speed (µm/s)	Acceleration (µm/s^2^)	Coordinate Position (µm)
300 kHz	C_1_	−5.67382	438.2029	30.86125
	C_2_	23.46077	−6672.94	182.3383
800 kHz	C_1_	−8.73413	−107.999	592.298
	C_2_	11.15337	−69.2376	657.8384
	C_3_	−5.78761	100.1757	699.9772
	C_4_	11.24438	−32.3256	763.6354
15 MHz	C_1_	28.33875	−5.31 × 10^−14^	52.33616
	C_2_	−22.9694	−1.84 × 10^−7^	270.3973

**Table 9:** The overall comparative data analysis of the experimental and the computational results of particles/cells in various frequency ranges.

Medium	Particles/Cells	MyDEP			Experimental Results			FEM Response		
		*P_DEP_*	*f_XO_*	*N_DEP_*	*P_DEP_*	*f_XO_*	*N_DEP_*	*P_DEP_*	*f_XO_*	*N_DEP_*
DI water- (Low conductive Medium)	Polystyrene particle 3.2 µm	*f* < 425.02 kHz	425.02 kHz	*f* > 425.02 kHz	*f* < 420 kHz	420 kHz	*f* > 420 kHz	*f* < 425 kHz	425 kHz	*f* > 425 kHz
	Polystyrene particle 4.8 µm	*f* < 275.37 kHz	275.37 kHz	*f* > 275.37 kHz	*f* < 260 kHz	260 kHz	*f* > 260 kHz	*f* < 275.013 kHz	275.013 kHz	*f* > 275.013 kHz
DI water- (Low conductive Medium)	Keratinocyte cell	*f* < 28.10 MHz	28.10 MHz	*f* > 28.10 MHz	-			*f* < 28.07 MHz	28.07 MHz	*f* > 28.07 MHz
	Fibroblast cells	*f* < 510.53 MHz	510.53 MHz	*f* > 510.53 MHz				*f* < 510.1 MHz	510.1 MHz	*f* > 510.1 MHz
DMEM/F12- (High conductive Medium)	Keratinocyte cell	N/A	N/A	All range only *N_DEP_* exist	N/A	N/A	All range only *N_DEP_* exist	N/A	N/A	All range only *N_DEP_* exist
DMEM-High conductive Medium	Fibroblast cells									

**Table 10:** Reported studies based on the keratinocyte and fibroblast cells using DEP.

Target Cells	Input Frequency	Efficiency/Supply Voltage
Separation of Human normal epithelial cells (HaCaT) from polystyrene beads [50]	f = 100 kHz to 5 MHz, *N_DEP_* (beads) f = 700 kHz. *f_XO_*(cell)	10 V_pp_, frequency sweep—100 kHz to 5 MHz, Continuous separation at 1 MHz.
Separation of Macrophages and Fibroblasts [51]	f = 20 kHz, 90% of fibroblasts trapped, macrophages trapped less than 20%.	At 350 V_rms_ and 1.25 µL/min
Separation of live and dead Swiss mouse fibroblast (NIH-3T3) cells [52]	f = 150 kHz or 300 kHz, separation between the live and dead cells	V_SEP_ = 3.4 V_PP_, 87.3% efficiency, Throughput~1302 cells/min
Characterize Canola plant protoplast and ligament fibroblast cells [53]	f = 1 kHz to 50 MHz (*P_DEP_* response for both cells).	C_eff_ = 0.47 ± 0.03, µF/cm^2^ (Protoplasts) C_eff_ = 1.52 ± 0.26 µF/cm^2^ (fibroblasts)
Manipulation of fibroblast (3T3) cells [54]	f = 50 kHz, CMF close to − 0.5 (separation)	AC voltage at 10 V_pp_, Frequency sweep—10 MHz and 50 kHz.
Characterize Normal keratinocytes (UP) versus oral squamous cell carcinoma cell lines (H357) [48]	f = 5 kHz, collected H357 cells from keratinocyte cell.	C_mem_ = (11.3 mF/m^2^) Cc_ytoplasm_ = 0.45 (S/m)
Manipulation of Human keratinocyte (Present work)	In low conductivity, f = 28.43 MHz (*f_XO_*, keratinocyte cell) f = 510.1MHz (*f_XO_*, Fibroblast cell)	Ac signal at 10 V_pp_, frequency sweep—10 kHz to 800 MHz

## Data Availability

The data presented in this study are available within this article.

## Supplementary Materials

The following are available online at https://www.mdpi.com/article/10.3390/s21093007/s1, Video S1: Figure 12a,b, Video S2: Figure 13c,d, Video S3: Figure 14a,b. The supplementary text document includes cell modelling formula and the 2D/3D motion analysis software DIPP-MotionV procedure for Section 2.6.
